# Kinetic Gait Parameters in Unilateral Lower Limb Amputations and Normal Gait in Able-Bodied: Reference Values for Clinical Application

**DOI:** 10.3390/jcm11102683

**Published:** 2022-05-10

**Authors:** Karin Schmid-Zalaudek, Theresa Fischer, Zoltán Száva, Helmut Karl Lackner, Ursula Kropiunig, Christian Bittner, Karl Höcker, Günther Winkler, Gerfried Peternell

**Affiliations:** 1 Physiology Division, Otto Loewi Research Center for Vascular Biology, Immunology and Inflammation, Medical University of Graz, 8010 Graz, Austria; helmut.lackner@medunigraz.at; 2 Information and Communication Technology Division, Austrian Workers’ Compensation Board (AUVA), 1100 Vienna, Austria; theresa.fischer@auva.at (T.F.); zoltan.szava@auva.at (Z.S.); 3 Rehabilitation Clinic Tobelbad, Austrian Workers’ Compensation Board (AUVA), 8144 Tobelbad, Austria; ursula.kropiunig@auva.at; 4 Rehabilitation Center Häring, Austrian Workers’ Compensation Board (AUVA), 6323 Bad Häring, Austria; christian.bittner@auva.at; 5 Rehabilitation Center Weißer Hof, Austrian Workers’ Compensation Board (AUVA), 3400 Klosterneuburg, Austria; karl.hoecker@auva.at (K.H.); guenther.winkler@auva.at (G.W.)

**Keywords:** kinetic gait parameters, spatiotemporal parameters, ground reaction force parameters lower limb amputations, transfemoral amputations, transtibial amputations, foot amputations, knee disarticulations, gait reference values

## Abstract

Unilateral lower limb amputations usually present with asymmetric interlimb gait patterns, in the long term leading to secondary physical conditions and carrying the risk of low physical activity and impairment of general health. To assess prosthetic fittings and rehabilitation measures, reference values for asymmetries as well as the most significant gait parameters are required. Kinetic gait data of 865 patients with unilateral lower limb amputations (hip and knee disarticulations, transfemoral, transtibial and foot amputations) and 216 able-bodied participants were quantitatively assessed by instrumented gait analyses. Characteristic spatiotemporal (stance time, walking speed, step length and width) and ground reaction force parameters (weight-acceptance and push-off peak) were contrasted to normal gait. All spatiotemporal and ground reaction force parameters differed significantly from normal gait with the largest differences in transfemoral amputations. These also differed between amputation levels and showed age-dependencies. The stance time and push-off peak difference were identified as the most discriminative parameters with the highest diagnostic specificity and sensitivity. The present results mark the first step to establishing universal reference values for gait parameters by means of which the quality and suitability of a prosthetic fitting and the rehabilitation progress can be assessed, and are generalizable for all adults with unilateral lower limb amputations in terms of level walking.

## 1. Introduction

Due to the loss of physiological joint and muscle functions and accompanying gait alterations, patients with unilateral lower limb amputations (ULLA) usually present asymmetric interlimb gait patterns and deviations from normal gait in spatiotemporal and ground reaction force (GRF) parameters [1,2,3,4,5,6,7,8,9,10,11,12,13,14,15,16,17,18,19]. The stance time of the prosthetic limb, for example, is shorter when compared to the intact limb, while the swing and step times are longer [1,3,4,5,7,8,11,12,14,17,18,19,20,21]. The stance time of the intact limb is even longer than the stance time presented by able-bodied people [11,14,21]. Additionally, the step length presents interlimb asymmetries, patient-dependent [9,14,22] either being longer [1,12,13,20,23] or shorter [7,18] for the prosthetic limb. The step width of patients with ULLA, on the contrary, is constantly higher when compared to able-bodied people [5,18,24], whereas their walking speed is generally reduced [2,3,7,15,22,25]. The weight-acceptance and the push-off peak of the vertical component of the ground reaction force (GRF) are higher on the intact limb [4,6,8,14,16,18,19,21], though, in patients with transfemoral amputations, the weight-acceptance peak might also be higher on the prosthetic limb [15,18]. Compared to the peaks of the able-bodied, they show the tendency to be smaller [4,6,19,21].

The occurrence and the amount of the asymmetries described depend on a series of factors, originating from either the prosthesis or the patient. These include the amputation level [18,26], the quality and length of the residual limb [2,10,27], the prosthetic components [12,13,15,18,22,26], the prosthetic alignment [28], the socket fit, the patient’s age, the reason for [25] and the time since amputation as well as the rehabilitation program. Considering the amputation level, a more asymmetrical gait pattern is found in transfemoral than in transtibial amputations, especially in terms of temporal parameters [4,7,15,18,26]. Additionally, the shorter the residual limb is, the more asymmetrical the gait pattern becomes [10,27]. Furthermore, asymmetries are also influenced by walking speed; whereas temporal asymmetries reduce, loading asymmetries increase at higher walking speeds [2,4,8,19]. Different kinds of technologies in terms of prosthetic components, such as energy storage and return prostheses, microprocessor controlled or bionic joints though have the potential to reduce asymmetries, at best up to an extent that is almost comparable to those of able-bodied people [12,18,22]. 

In the long term, the asymmetries between the intact and the prosthetic limb and the excessive loading of the intact limb might lead to secondary physical conditions, such as joint and bone degenerations or lower back pain, carrying the risk of low physical activity and subsequent impairment of general health [4,29,30,31,32]. Especially, an increased prevalence of knee osteoarthritis is observed [31,33]. Therefore, people with ULLA are encouraged to strive for the best possible symmetric gait pattern. Yet, certain asymmetries remain unavoidable due to the persistent structural differences in the neuromuscular and skeletal systems [34]. For the evaluation of rehabilitation measures and the selection of the best prosthetic fitting, it is thus necessary to define universal reference values [15] for the non-symmetrical optimal [34]. With the availability of profound reference values, entering the clinical practice, it can be determined what a desirable gait pattern should look like. Furthermore, the likelihood of the occurrence of physical consequences further impairing the patient’s health and quality of life as well as the medical expenses of the secondary medical conditions might noticeably be reduced. Several attempts to define normative data [35] and symmetry indices [4,12] have already been made, though mainly based upon small sample sizes with strict inclusion and exclusion criteria and focusing on homogeneous patient collectives or certain amputation levels, limiting the generalizability and clinical applicability of the results.

Within the rehabilitation centers of the Austrian Worker’s Compensation Board (AUVA) an instrumented gait analysis system is used to monitor the rehabilitation progress and to quantitatively assess the fitting of medical aids in the prosthetic and orthotic field. The AUVA, therefore, holds a large amount of kinetic gait data of patients and able-bodied people. The aim of the present study was hence to retrospectively analyze the large data set to determine characteristic asymmetry ranges in spatiotemporal and GRF parameters at different amputation levels and to contrast them to normal gait. Based on the aforementioned literature [28,36], several spatiotemporal and loading parameters are more likely to account for an acceptable and symmetric gait pattern, thus primarily the following were included: stance time, step length, step width, walking speed, cadence as well as weight-acceptance peak and push-off peak. Furthermore, among these, the parameter with the highest significance for the assessment of a symmetrical gait pattern should be identified. Focusing on a heterogeneous patient collective, the effects of prosthetic components, the prosthetic alignment, and the reason for and the time since amputation were deliberately not taken into consideration. 

## 2. Materials and Methods

### 2.1. Participants

Kinetic gait data of 865 patients with ULLA, ranging from 16 to 83 years (M = 49.73 ± 14.74), routinely collected during their inpatient rehabilitation stays, were enrolled. The amputation levels included were hip disarticulation (HDA), transfemoral amputation (TFA), knee disarticulation (KDA), transtibial amputation (TTA) and foot amputation (FA). Additionally, kinetic gait data of 216 able-bodied voluntary participants, free from any serious injuries to the lower limbs, were included. 

### 2.2. Data Collection

The three-dimensional, instrumented gait analyses were performed in the AUVA’s gait laboratories following the standardized protocol of the ‘Applied Gait Analysis’ [37]. Each laboratory is equipped with at least two piezoelectric force plates (Kistler Instrumente AG, Winterthur, Switzerland), embedded in the middle of a 10 m level walkway, operating at 2000 Hz. The patients walked up and down the walkway at a self-paced walking speed, wearing athletic shoes, until ten correct trials were recorded. Trials, during which the patients stepped outside the plates’ edges or loaded two plates at the same time, were excluded. Since the prosthetic fitting is adapted several times during an inpatient stay, usually the latest measurement result of each patient was included in the study. 

### 2.3. Data Preprocessing

GRF data were analyzed with MATLAB (The MathWorks, Inc., Natick, MA, USA). Prior to the calculation of spatiotemporal and GRF parameters, data were sampled down to 250 Hz and normalized to the patient’s body mass at measurement time. A threshold of 100 N of the resultant force was used to determine the gait events’ initial contact (IC) and toe off (TO). The spatiotemporal and GRF parameters calculated were stance time, step length, step width, walking speed, cadence, weight-acceptance and push-off peak [38]. Homogeneity of walking was defined by the absolute difference between the intact and the prosthetic limb, or in able-bodied, between the left and right leg.

### 2.4. Statistical Analysis

Based on the given study design, retrospectively analyzed collected data, an a priori sample size calculation was dispensed. However, we calculated the partial η^2^ coefficient in the ANOVAs to estimate the effect and variance explained by the amputation level. All data were explored regarding their distribution and outliers largely deviating from the respective group mean (mean ± 3SD) were excluded. The frequency of amputation levels was compared by χ^2^ tests. Kinetic gait parameters of patients, grouped by amputation level, and able-bodied participants were analyzed by Welch-ANOVA accounting for large sample size differences and Games–Howell post-hoc tests to compare the subgroups. Additionally, a multivariate linear regression including the asymmetry of stance time, step length, weight acceptance peak and push-off peak and step width as predictors was applied to analyze the influence of gait homogeneity on walking speed. Furthermore, a ROC (receiver operating characteristics) analysis was performed to assess the sensitivity and specificity of the parameters and graphically plot their diagnostic value. Due to the age-dependency of gait parameters [39,40], patients younger than 15 years were excluded and older patients were analyzed separately, splitting the group by mean age at retirement (60 years). All data were analyzed by IBM SPSS Statistics for Windows (IBM Corp., Armonk, NY, USA) considering a *p*-value of α < 0.05 as significant. Due to the low prevalence of HDA (*n* = 22, 12 using walking aids), patients of this group were not included in the analyses, as were patients using any kind of walking aids (Table 1). For the purpose of comparison though, mean values are shown in tables and figures.

## 3. Results

The total sample consisted of 699 male and 166 female patients with ULLA (mean age = 49.73 ± 14.74) and 216 able-bodied subjects (M = 34.89 ± 14.01; 48% females). TFA (40.5%) and TTA (41.8%) were the most frequent amputations in both, females (χ^2^_(4)_ = 98.52, *p* < 0.001) and males (χ^2^_(4)_ = 557.45, *p* < 0.001). Patients using walking aids were on average older (M = 56.54 ± 14.9; *n* = 227) than patients without walking aids (M = 47.73 ± 14.9; *n* = 638, t_(863)_ = −8.32, *p* < 0.001), and the use of walking aids was most prevalent in HDA and TTA (χ^2^ = 14.81, *p* = 0.005) (Table 1). 

**Table 1:** Prevalence of amputation levels split by age and sex.

		Amputation Level					
		HDA	TFA	KDA	TTA	FA	Total
*<60 years*	female	9	50	14	48	9	130
		6.9%	38.5%	10.8%	36.9%	6.9%	100.0%
	male	11	232	40	204	49	536
		2.1%	43.3%	7.5%	38.1%	9.1%	100.0%
	total	20	282	54	252	58	666
		3.0%	42.3%	8.1%	37.8%	8.7%	100.0%
*>60 years*	female	2	14	0	17	3	36
		5.6%	38.9%	0.0%	47.2%	8.3%	100.0%
	male	0	54	10	93	6	163
		0.0%	33.1%	6.1%	57.1%	3.7%	100.0%
	total	2	68	10	110	9	199
		1.0%	34.2%	5.0%	55.3%	4.5%	100.0%
total		22 (2.5%)	350 (40.5%)	64 (7.4%)	362 (41.8%)	67 (7.7%)	865
use of walking aids		12	92	10	100	13	227
(*excluded*)		54.5%	26.3%	15.6%	27.6%	19.4%	26.2%
Total included < 60 years		9	222	48	208	49	536 *
Total included > 60 years		1	36	6	54	5	103 *

Abbreviations: HDA: hip disarticulation, TFA: transfemoral amputation, KDA: knee disarticulation, TTA: transtibial amputation, FA: foot amputation; * after exclusion of outliers and patients using walking aids.

### 3.1. Kinetic Gait Parameters

The mean walking speed differed significantly between the patient groups (F_(4, 169)_ = 40.78, *p* < 0.001, η^2^ = 0.161) and was lowest in TFA followed by FA (Table 2). All patients walked significantly slower than able-bodied subjects (*p* < 0.001). Patients with TFA showed a lower walking speed compared to KDA (*p* = 0.039) and TTA (*p* = 0.002), but not compared to FA (*p* = 0.959). No significant difference was found between KDA and TTA (*p* = 0.983) or FA (*p* = 0.660) nor between TTA and FA (*p* = 0.788). 

The mean cadence was significantly lower in all patient groups (F_(4, 173)_ = 46.01, *p* < 0.001, η^2^ = 0.197), with the lowest values in TFA and FA (Table 2). Additionally, cadence in TFA was lower compared to KDA (*p* = 0.015) and TTA (*p* = 0.011), but not compared to FA (*p* = 0.438). 

The step width was significantly larger in all patient groups (F_(4, 170)_ = 164.73, *p* < 0.001, η^2^ = 0.471), with the largest step width at the highest amputation level (Table 2). Step width in TFA was significantly larger compared to TTA (*p* < 0.001) and FA (*p* < 0.001) as well as in KDA compared to TTA (*p* = 0.004) and FA (*p* = 0.023). No difference was found between TTA and FA.

**Figure 1:**
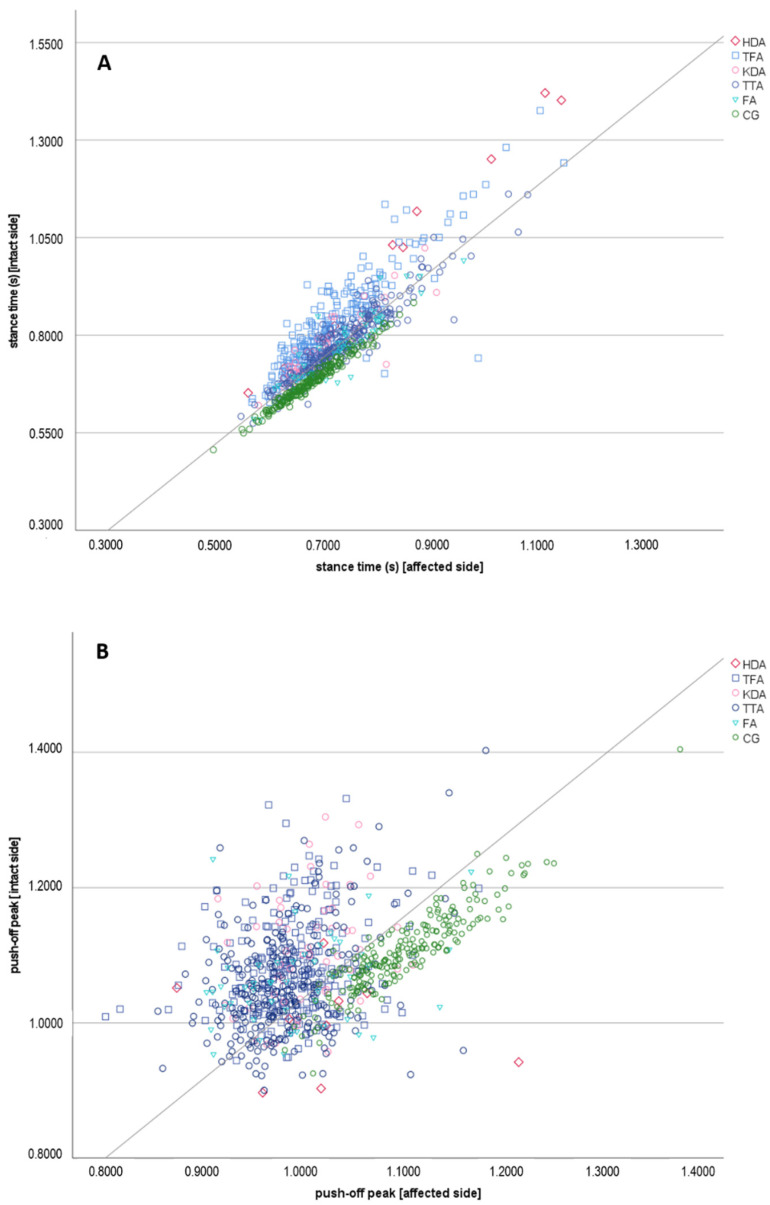
(**A**): Stance time difference between the intact and affected limb at different amputation levels and in healthy participants (left/right side, resp.) (**B**): Push-off peak difference between the intact and affected limb at different amputation levels and in healthy participants (left/right side, resp.).

Similarly, the stance time difference was significantly larger in all patient groups (F_(4, 156)_ = 280.70, *p* < 0.001, η^2^ = 0.522), with TFA showing the largest difference (Table 2). All patient groups also differed from TFA (*p* < 0.001), though there was no statistical difference between KDA, TTA and FA. Figure 1 (upper part) displays the stance time in able-bodied people contrasted to different amputation levels (Figure 1A). Comparison of the mean stance time indicated a significantly shorter stance time of the amputated limb in TFA (t_(217)_ = 24.09, *p* < 0.001), KDA (t_(47)_ = 12.25, *p* < 0.001), TTA (t_(203)_ = 18.19, *p* < 0.001) and FA (t_(46)_ = 6.15, *p* < 0.001) (Table 2).

The step length difference was significantly larger in all patients (F_(4, 165)_ = 561.40, *p* < 0.001), with the largest difference in FA, and a larger difference in TFA than in TTA (*p* = 0.030). The comparison of sides indicated a larger step length of the amputated side in all patient groups, which was significant in TFA (t_(214)_ = −3.364, *p* < 0.001) and FA (t_(45)_ = −4.81, *p* < 0.001).

Considering the weight-acceptance peak, all patients showed a larger difference than the able-bodied subjects (F_(4, 158)_ = 96.08, *p* < 0.001, η^2^ = 0.209) with the largest difference in the FA group (Table 2), but no differences between the patient groups. 

Also the absolute push-off peak difference was significantly larger in all patients (F_(4, 157)_ = 151.45, *p* < 0.001, η^2^ = 0.276) with the largest asymmetry in KDA, while no differences were found between the patients groups (Table 2). Furthermore, the comparison of the amputated and intact limb revealed a larger weight-acceptance peak of the intact limb in TFA (t_(217)_ = 6.49, *p* < 0.001), KDA (t_(47)_ = 1.71, *p* < 0.047), TTA (t_(203)_ = 11.72, *p* < 0.001) and FA (t_(46)_ = 6.13, *p* < 0.001). Additionally, the mean push-off peak was larger for the intact limb in TFA (t_(217)_ = 17.34, *p* < 0.001), KDA (t_(47)_ = 8.91, *p* < 0.001), TTA (t_(207)_ = 14.72, *p* < 0.001) and FA (t_(46)_ = 5.67, *p* < 0.001) (Figure 1B).

### 3.2. Parameters for a Symmetrical Gait Pattern

Regarding the results of the multivariate linear regression, a significant model (F_(5, 207)_ = 3.82, *p* = 0.003) was observed in the able-bodied group, indicating the asymmetry of the push-off peak as significantly contributing to walking speed, though the overall fit was low (R^2^ = 0.294, R^2^adj. = 0.086, Table 3). In the patients’ group, a significant model (F_(5, 505)_ = 51.10, *p* < 0.001) was observed indicating stance time asymmetry as the best predictor for walking speed, followed by the push-off peak (R^2^ = 0.338, R^2^adj. = 0.332) with a high goodness of fit (Table 3A). Multivariate regression was also performed group-wise and confirmed the stance time and push-off peak difference as the most important predictors in all groups.

**Figure 2:**
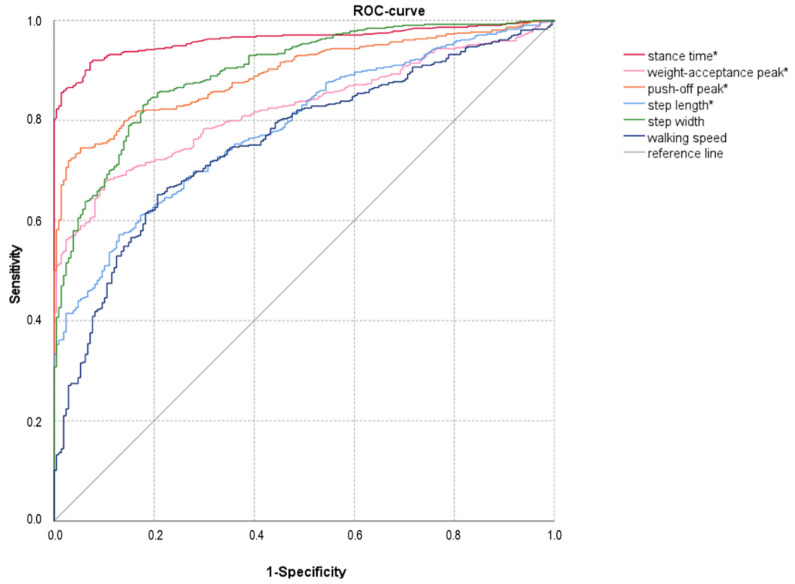
Sensitivity and specificity of kinetic gait parameters in patients with ULLA (* difference between intact and affected side).

The ROC analysis displays the diagnostic accuracy and very high sensitivity of the stance time (AUC = 0.962, *p* < 0.001) and the push-off peak (AUC = 0.894, *p* < 0.001) difference compared to other gait characteristics (Table 3B). All patients/participants were correctly classified, and by applying a cut-off value of 0.01733 s for stance time difference a maximum sensitivity (0.92) and specificity (0.93) can be achieved with the highest Youden-index (sensitivity + specificity − 1) at J = 0.84 (Figure 2).

### 3.3. Kinetic Gait in Older Patients

Due to the low prevalence of HDA, KDA and FA in elderly patients (Table 1), only patients with TFA and TTA were statistically compared to the corresponding group of younger patients. For TFA, a multivariate ANOVA revealed a significant effect between older and younger patients (F_(6, 242)_ = 8.583, *p* < 0.001, η^2^ = 0.175). The stance time (F_(1, 242)_ = 12.86, *p* < 0.001, η^2^ = 0.05) and step length (F_(1, 242)_ = 4.90, *p* = 0.028, η^2^ = 0.019) differences became larger, the step width broader (F_(1, 242)_ = 4.15, *p* = 0.043, η^2^ = 0.017) and the walking speed slower (F_(1, 242)_ = 46.93, *p* < 0.001, η^2^ = 0.16) with age. Yet, the push-off peak difference was lower in elderly patients (F_(1, 242)_ = 46.93, *p* < 0.001, η^2^ = 0.16). In TTA the significant effect of age (F_(6, 241)_ = 9.809, *p* < 0.001, η^2^ = 0.196) was based on a significantly slower walking speed (F_(1, 241)_ = 46.57, *p* < 0.001, η^2^ = 0.159), while the asymmetries of the weight-acceptance (F_(1, 242)_ = 8.45, *p* = 0.004, η^2^ = 0.033) and push-off peak (F_(1, 242)_ = 20.62, *p* < 0.001, η^2^ = 0.077) were even smaller in elderly patients (Table 4).

## 4. Discussion

The results of the spatiotemporal and GRF parameters of people with ULLA correspond to the asymmetries reported in the literature [1,2,3,4,5,6,7,8,9,10,11,12,13,14,15,16,17,18,19]. The steady decrease of walking speed, cadence and increase of step width with amputation level (Table 2) indicate that these parameters are linked to the length of the residual limb [2,7,18] and substantiate that a patient is more unsteady the more joints are missing [41]. This is further proven by the absolute stance time difference, which shows a remarkable leap in value when FA, TTA and KDA are compared to HDA and TFA.

Considering the GRF peaks, it shows that the intact limb is loaded excessively during weight-acceptance, especially in FA, and less during push off. Yet, this does not apply to KDA, as their results are comparable to physiological gait. This might also explain why the absolute differences of the push-off peak are surprisingly high. The peaks of the amputated limb are smaller than the corresponding peaks of the abled-bodied participants. Whereas in terms of weight-acceptance, KDA and FA show, respectively, high peaks compared to TFA and TTA, only patients with KDA manage to slightly exceed their own body weight at the end of the stance phase. 

These findings also outline the importance of the load-bearing capacity of the residual limb and the potential advantages of prosthetic restorations in the form of joint disarticulations. They contradict the repeatedly postulated problematic effect of the height difference between the knee joint axes [42,43] and support the assumption, that people with KDA are less likely to be affected by pain than people with TFA or TTA [44]. As no differentiation between Lisfranc, Chopart, Pirogoff, or Syme 47 was recorded for FA, no further conclusive findings could be drawn in this regard. Yet, the variability of the results allows the presumption that the amputation level and the accompanying soft tissue coverage influence the load-bearing capacity distinctively.

The patient’s age has a modulating role on most spatiotemporal gait parameters, indicating that gait becomes slower and more asymmetrical with age, while asymmetries of the loading parameters do not significantly change with age. 

According to the results of the multivariate linear regression and ROC analysis, the parameter with the highest sensitivity for a simple assessment of the homogeneity of gait is the stance time (difference), followed by the push-off peak. Physical interventions or adjustments thus should emphasize the improvement of the symmetry of these parameters, to prevent non-use of prostheses and further deterioration [45]. 

The present study differentiates from previously conducted studies most notably in terms of the number of underlying data. Moreover, it did not focus on any specific kinds of prosthetic components, fittings, or technologies [12,15] nor did it take the reason for or the time since amputation into consideration. Therefore, the results are to the greatest possible extent generalizable for all adults with ULLA, allowing intra and interindividual comparisons of absolute values in common units. 

Yet, it has to be considered that the reference values proposed are only valid for level walking and are not applicable to uphill or downhill walking. Especially the preservation of an intact knee joint is keen for the latter, and therefore, it can be assumed that the asymmetry ranges will then be more pronounced. Furthermore, the low number of patients with HDA, especially older patients and without the usage of walking-aids, limits the applicability and evaluation of gait patterns in this patient group. Another limitation concerns the sampling method, using data routinely assessed during the inpatient stay, which might bias the results and impact the power.

## 5. Conclusions

The universal reference values for interlimb gait patterns of patients with ULLA provide physicians, orthopedic technicians, therapists and researchers with a way to evaluate any kind of changes to the prosthetic alignment and to assess innovations in the connection between the residual limb and the socket or the success of a particular gait training. Furthermore, they support the quantified documentation of rehabilitation measures, add to the standardization of the assessment of prosthetic fittings and might even be used to test the postulated effect of prosthetic components to justify their use by the payers. The resulting asymmetry ranges should provide a profound basis by means of which it could be determined how a desirable gait pattern looks when all guidelines on rehabilitation post amputation are thoroughly followed. 

## Figures and Tables

**Table 2:** Reference values for kinetic gait parameters in unilateral lower limb amputations and physiological gait (age: 16–60 years).

		HDA (*n* = 9)		TFA (*n* = 222)		KDA (*n* = 48)		TTA (*n* = 208)		FA (*n* = 49)		NORM (*n* = 201)	
Age	mean ± SD	35.13	±12.46	44.31	±11.07	41.63	±12.42	43.64	±12.08	42.89	±11.59	33.94	±12.94
	95% CI	24.71	45.54	42.82	45.79	37.94	45.32	41.95	45.33	39.45	46.33	32.17	35.71
	min-max	17	49	16	60	16	60	16	60	16	59	16	60
Walking speed **	mean ± SD	55.62	±13.87	66.15	±8.80	70.34	±8.89	69.53	±10.29	67.40	±11.73	76.23	±7.90
(m/min)	95% CI	44.03	67.22	64.97	67.34	67.70	72.98	68.09	70.97	63.92	70.89	75.15	77.32
	median	52.61		66.05		70.23		68.85		66.00		76.28	
	min-max	39.69	79.08	41.75	89.65	52.57	96.37	39.13	98.80	46.58	103.20	55.66	100.92
Cadence **	mean ± SD	87.7095	±19.4387	100.7493	±9.4313	105.0909	±7.9803	103.7823	±9.4822	103.7325	±11.0224	112.2100	±8.6769
(1/min)	95% CI	71.4583	103.9606	99.4815	102.0172	102.7210	107.4607	102.4568	105.1079	100.4592	107.0058	111.0238	113.3961
	median	83.5410		101.4885		105.6078		103.8062		102.7089		112.4227	
	min-max	63.5055	119.0476	72.4813	122.7496	87.2995	122.0504	68.1818	135.0193	81.8889	131.9261	88.1597	141.9558
Step width (m) **	mean ± SD	0.1565	±0.0209	0.1651	±0.0350	0.1503	±0.0345	0.1299	±0.0286	0.1293	±0.0310	0.0921	±0.0251
	95% CI	0.1390	0.1741	0.1604	0.1698	0.1400	0.1605	0.1259	0.1339	0.1201	0.1385	0.0887	0.0956
	median	0.1591		0.1646		0.1435		0.1274		0.1213		0.0915	
	min-max	0.1250	0.1856	0.0792	0.2970	0.0875	0.2424	0.0499	0.2190	0.0785	0.2185	0.0271	0.1631
Stance time (s) **	mean ± SD	1.0047	±0.2642	0.8070	±0.1026	0.7581	±0.0708	0.7704	±0.0853	0.7718	±0.0952	0.6819	±0.0599
intact limb	95% CI	0.7839	1.2256	0.7932	0.8208	0.7371	0.7791	0.7585	0.7823	0.7435	0.8001	0.6737	0.6901
	median	1.0283		0.7832		0.7489		0.7607		0.7698		0.6768	
	min-max	0.6520	1.4208	0.6276	1.1860	0.6204	0.9531	0.5744	1.1618	0.5836	0.9920	0.5063	0.8834
Stance time (s) **	mean ± SD	0.8309	±0.1880	0.7157	±0.0780	0.7054	±0.0647	0.7301	±0.0767	0.7373	±0.0831	0.6833	±0.0603
amp. limb	95% CI	0.6737	0.9881	0.7052	0.7262	0.6861	0.7246	0.7194	0.7409	0.7127	0.7620	0.6751	0.6916
	median	0.8467		0.7024		0.6973		0.7196		0.7407		0.6773	
	min-max	0.5644	1.1244	0.5713	1.0124	0.5836	0.9196	0.5508	1.0553	0.5748	0.9710	0.4987	0.8766
Stance time (s) **	mean ± SD	0.1738	±0.0791	0.0952	±0.0495	0.0533	±0.0292	0.0432	±0.0273	0.0410	±0.0317	0.0076	±0.0059
*diff.*	95% CI	0.1077	0.2399	0.0886	0.1019	0.0447	0.0620	0.0394	0.0471	0.0316	0.0504	0.0068	0.0084
	median	0.1816		0.0898		0.0555		0.0382		0.0336		0.0062	
	min-max	0.0831	0.2964	0.0173	0.2580	0.0003	0.1204	0.0006	0.1492	0.0022	0.1530	0.0000	0.0280
Weight-acceptance **	mean ± SD	1.1531	±0.1291	1.1151	±0.0997	1.1084	±0.1080	1.1343	±0.0864	1.2036	±0.1369	1.1162	±0.0702
peak (intact limb)	95% CI	1.0451	1.2610	1.1017	1.1285	1.0763	1.1405	1.1222	1.1464	1.1629	1.2442	1.1066	1.1258
	median	1.0991		1.1153		1.0968		1.1247		1.1661		1.1099	
	min-max	1.0558	1.4027	0.8761	1.4013	0.9307	1.4258	0.9364	1.4288	0.9591	1.6149	0.9971	1.3307
Weight-acceptance **	mean ± SD	1.1552	±0.0891	1.0632	±0.0850	1.0807	±0.0725	1.0607	±0.0709	1.0980	±0.0904	1.1200	±0.0691
peak (amp. limb)	95% CI	1.0807	1.2297	1.0517	1.0746	1.0592	1.1022	1.0508	1.0707	1.0712	1.1249	1.1106	1.1295
	median	1.1415		1.0468		1.0802		1.0529		1.0784		1.1093	
	min-max	1.0619	1.3083	0.8487	1.4693	0.9478	1.2527	0.9130	1.3253	0.9664	1.4150	0.9955	1.3267
Weight-acceptance **	mean ± SD	0.0604	±0.0572	0.1006	±0.0805	0.0905	±0.0706	0.0921	±0.0696	0.1298	±0.0930	0.0268	±0.0193
peak (*diff.*)	95% CI	0.0126	0.1082	0.0897	0.1114	0.0695	0.1114	0.0824	0.1018	0.1022	0.1575	0.0241	0.0294
	median	0.0483		0.0860		0.0749		0.0763		0.1159		0.0229	
	min-max	0.0047	0.1588	0.0007	0.3974	0.0003	0.2822	0.0013	0.3198	0.0095	0.3921	0.0000	0.1000
Push-off peak **	mean ± SD	0.9924	±0.0755	1.0842	±0.0722	1.1130	±0.0805	1.0673	±0.0811	1.0653	±0.0683	1.1078	±0.0588
(intact limb)	95% CI	0.9293	1.0555	1.0745	1.0939	1.0891	1.1369	1.0559	1.0786	1.0450	1.0856	1.0998	1.1159
	median	1.0012		1.0736		1.1002		1.0557		1.0566		1.1035	
	min-max	0.8970	1.1183	0.9442	1.3314	0.9682	1.3044	0.9005	1.4026	0.9537	1.2420	0.9254	1.4044
Push-off peak **	mean ± SD	1.0440	±0.0793	0.9925	±0.0472	1.0064	±0.0457	0.9858	±0.0555	0.9972	±0.0653	1.1080	±0.0569
(amp. limb)	95% CI	0.9777	1.1103	0.9862	0.9989	0.9928	1.0200	0.9781	0.9936	0.9779	1.0166	1.1003	1.1158
	median	1.0251		0.9910		1.0084		0.9760		0.9891		1.1004	
	min-max	0.9613	1.2235	0.8003	1.1826	0.9155	1.1151	0.8540	1.1898	0.9038	1.1748	0.9840	1.3888
Push-off peak **	mean ± SD	0.0797	±0.0905	0.0993	±0.0675	0.1106	±0.0779	0.0941	±0.0649	0.0876	±0.0626	0.0199	±0.0149
(*diff.*)	95% CI	0.0040	0.1553	0.0903	0.1084	0.0875	0.1338	0.0850	0.1032	0.0690	0.1062	0.0178	0.0219
	median	0.0472		0.0900		0.0929		0.0827		0.0865		0.0152	
	min-max	0.0068	0.2814	0.0017	0.3546	0.0027	0.2789	0.0001	0.3410	0.0060	0.3316	0.0000	0.0875
Step length (m) **	mean ± SD	0.6255	±0.0363	0.6442	±0.0524	0.6619	±0.0557	0.6649	±0.0605	0.6272	±0.0670	0.6778	±0.0375
intact limb	95% CI	0.5952	0.6559	0.6372	0.6513	0.6453	0.6784	0.6564	0.6733	0.6073	0.6471	0.6726	0.6829
	median	0.6303		0.6457		0.6650		0.6615		0.6153		0.6760	
	min-max	0.5496	0.6665	0.5161	0.7992	0.5387	0.8464	0.5070	0.9126	0.5195	0.8636	0.5797	0.8103
Step length (m) **	mean ± SD	0.6373	±0.0319	0.6573	±0.0508	0.6710	±0.0668	0.6689	±0.0615	0.6653	±0.0611	0.6789	±0.0380
amp. limb	95% CI	0.6106	0.6640	0.6504	0.6641	0.6512	0.6909	0.6603	0.6775	0.6471	0.6834	0.6737	0.6841
	median	0.6408		0.6572		0.6698		0.6675		0.6617		0.6756	
	min-max	0.5960	0.6830	0.5423	0.8664	0.5280	0.8843	0.5363	0.8344	0.5597	0.9021	0.5872	0.8237
Step length (m) **	mean ± SD	0.0392	0.0168	0.0477	0.0334	0.0456	0.0283	0.0392	0.0278	0.0528	0.0390	0.0173	0.0133
*diff.*	95% CI	0.0252	0.0533	0.0432	0.0522	0.0372	0.0540	0.0353	0.0431	0.0412	0.0644	0.0155	0.0192
	median	0.0447		0.0404		0.0440		0.0331		0.0470		0.0147	
	min-max	0.0097	0.0552	0.0004	0.1549	0.0009	0.1059	0.0000	0.1287	0.0021	0.1544	0.0001	0.0539

Abbreviations: HDA: hip disarticulation, TFA: transfemoral amputation, KDA: knee disarticulation, TTA: transtibial amputation, FA: foot amputation, NORM: normal gait (control group); *amp. limb*: amputated limb, *diff.*: difference between amputated and intact limb (correspondingly left/right limb in able-bodied); Note: *walking speed*: describes how fast a person moves in m/min; *cadence*: number of steps per minute; *step width*: distance between the heels of the two feet during double stance; *stance time*: time between the initial contact (IC) and the following toe-off (TO) of the same foot (time during which the foot has ground contact); *weight-acceptance peak and push-off peak*: the two characteristic peaks of the typically M-shaped vertical component of the GRF, the first taking place at the transition from loading response to mid stance, the second during terminal stance; *step length*: the spatial distance from the IC of one foot to the IC of the contralateral foot. Significant differences between normal gait in healthy subjects and patients are indicated by ** *p* < 0.001, and similarly in patients between the intact and prosthetic side.

**Table 3:** Regression analysis summary of kinetic gait parameters predicting walking speed (**A**) and summary of ROC analysis (**B**).

**(A)**	**Variable**	**B**	**SE**	***β***	**t**	***p***
*patients*	(constant)	70.69	1.66		47.71	<0.001
	stance time *	−86.17	8.3	−0.405	−10.39	<0.001
	weight−acceptance peak *	10.55	4.94	0.083	2.14	0.033
	push−off peak *	47.91	5.69	0.327	8.42	<0.001
*controls*	(constant)	75.9	2.49		30.51	<0.001
	push−off peak *	113.13	36.32	0.213	3.11	0.002
**(B)**		**AUC**	**SE**	***p***	***CI***	
					**Lower**	**Upper**
stance time * [s]		0.962	0.007	0.000	0.948	0.975
weight acceptance peak *		0.824	0.015	0.000	0.795	0.853
push−off peak *		0.894	0.011	0.000	0.871	0.916
step length * [m]		0.786	0.017	0.000	0.753	0.819
walking speed [m/min]		0.757	0.019	0.000	0.721	0.794
step width [m]		0.897	0.012	0.000	0.874	0.92

Note: * indicates the difference between the affected and intact limb (correspondingly the left and right leg in healthy controls) in the gait parameters.

**Table 4:** Reference values for kinetic gait parameters in unilateral lower limb amputations and physiological gait (age > 60 years).

		TFA (*n* = 36)		KDA (*n* = 6)		TTA (*n* = 54)		FA (*n* = 5)		NORM (*n* = 6)	
Age	mean ± SD	67.53	±4.62	67.00	±6.48	67.96	±5.87	71.00	±4.24	65.00	±2.16
	95% CI	65.92	69.14	60.20	73.80	66.27	69.64	32.88	109.12	61.56	68.44
	median	67		63.5		67		71		65.5	
	min−max	61	78	63	79	61	83	68	74	62	67
Walking speed	mean ± SD	55.03	±8.77 **	62.18	±7.77	58.70	±8.43 **	58.19	±0.59	69.56	±2.43
(m/min)	95% CI	51.97	58.09	54.03	70.34	56.28	61.12	52.93	63.45	65.69	73.44
	median	56.65		64.19		59.66		58.19		69.95	
	min−max	35.51	69.09	48.56	69.75	38.80	76.70	57.77	58.60	66.26	72.09
Cadence	mean ± SD	90.8833	±11.0952 **	96.4483	±9.5907	95.4255	±9.5375	97.2344	±3.0366	112.9120	±5.2382
(1/min)	95% CI	87.0120	94.7546	86.3835	106.5131	92.6860	98.1650	69.9516	124.5172	104.5769	121.2471
	median	92.0238		98.2322		96.6270		97.2344		111.3532	
	min−max	63.2022	110.8647	79.2602	107.2194	70.8343	111.9299	95.0872	99.3816	108.4599	120.4819
Step width (m)	mean ± SD	0.1781	±0.0313 **	0.1783	±0.0291	0.1325	±0.0281	0.1410	±0.0001	0.1041	±0.0118
	95% CI	0.1672	0.1890	0.1478	0.2087	0.1244	0.1406	0.1404	0.1415	0.0853	0.1229
	median	0.1725		0.1792		0.1323		0.1410		0.1087	
	min−max	0.1214	0.2454	0.1397	0.2272	0.0700	0.1962	0.1409	0.1410	0.0867	0.1124
Stance time (s)	mean ± SD	0.9245	±0.1566 **	0.8374	±0.1145	0.8373	±0.0987	0.7999	±0.0189	0.6753	±0.0427
intact limb	95% CI	0.8698	0.9791	0.7172	0.9575	0.8089	0.8656	0.6298	0.9701	0.6075	0.7432
	median	0.9018		0.8257		0.8124		0.7999		0.6816	
	min−max	0.6964	1.3756	0.7232	1.0236	0.7080	1.1600	0.7866	0.8133	0.6177	0.7204
Stance time (s)	mean ± SD	0.7954	±0.1230	0.7730	±0.0782	0.7998	±0.0914	0.7923	±0.0199	0.6729	±0.0385
amp. limb	95% CI	0.7525	0.8383	0.6910	0.8551	0.7736	0.8261	0.6134	0.9711	0.6116	0.7343
	median	0.7818		0.7524		0.7893		0.7923		0.6767	
	min−max	0.6088	1.1596	0.6900	0.8976	0.6788	1.0916	0.7782	0.8063	0.6223	0.7160
Stance time (s)	mean ± SD	0.1291	±0.0606	0.0975	±0.0357	0.0399	±0.0245	0.0077	±0.0010	0.0047	±0.0014
*diff.*	95% CI	0.1079	0.1502	0.0601	0.1349	0.0328	0.0469	−0.0010	0.0164	0.0025	0.0069
	median	0.1181		0.1086		0.0360		0.0077		0.0045	
	min−max	0.0320	0.3128	0.0332	0.1260	0.0028	0.0916	0.0070	0.0084	0.0033	0.0065
Weight−acceptance	mean ± SD	1.0618	±0.0946	1.0866	±0.0966	1.0831	±0.0914	1.1082	±0.0142	1.0863	±0.0363
peak (intact limb)	95% CI	1.0288	1.0948	0.9852	1.1880	1.0569	1.1094	0.9805	1.2358	1.0286	1.1440
	median	1.0280		1.0921		1.0622		1.1082		1.0868	
	min−max	0.9630	1.3374	0.9667	1.1798	0.8745	1.3413	1.0981	1.1182	1.0506	1.1212
Weight−acceptance	mean ± SD	1.0440	±0.0695	1.0610	±0.0687	1.0576	±0.0584	1.0374	±0.0042	1.0983	±0.0426
peak (amp. limb)	95% CI	1.0197	1.0682	0.9890	1.1331	1.0408	1.0743	0.9993	1.0755	1.0306	1.1660
	median	1.0374		1.0269		1.0641		1.0374		1.0962	
	min−max	0.9122	1.2084	0.9971	1.1620	0.9233	1.2258	1.0344	1.0404	1.0484	1.1524
Weight−acceptance	mean ± SD	0.0741	±0.0709	0.0961	±0.0855	0.0608	±0.0573 **	0.0708	±0.0100	0.0228	±0.0157
peak (*diff.*)	95% CI	0.0493	0.0989	0.0064	0.1859	0.0444	0.0773	−0.0188	0.1603	−0.0023	0.0478
	median	0.0504		0.0931		0.0417		0.0708		0.0253	
	min−max	0.0021	0.2874	0.0070	0.1954	0.0001	0.2515	0.0637	0.0778	0.0022	0.0382
Push−off peak	mean ± SD	1.0535	±0.0512 **	1.0319	±0.0737	1.0189	±0.0528	1.0252	±0.0493	1.0445	±0.0635
(intact limb)	95% CI	1.0357	1.0714	0.9546	1.1092	1.0037	1.0341	0.5818	1.4686	0.9435	1.1456
	median	1.0462		1.0106		1.0221		1.0252		1.0282	
	min−max	0.9824	1.1912	0.9574	1.1362	0.9221	1.1283	0.9903	1.0601	0.9873	1.1345
Push−off peak	mean ± SD	0.9834	±0.0443	0.9924	±0.0438	0.9845	±0.0423	0.9387	±0.0429	1.0554	±0.0466
(amp. limb)	95% CI	0.9679	0.9988	0.9464	1.0385	0.9723	0.9966	0.5537	1.3237	0.9813	1.1295
	median	0.9888		0.9908		0.9831		0.9387		1.0440	
	min−max	0.8647	1.1115	0.9225	1.0477	0.8589	1.0731	0.9084	0.9690	1.0143	1.1194
Push−off peak	mean ± SD	0.0712	±0.0595	0.0648	±0.0510	0.0502	±0.0388 **	0.0865	±0.0065	0.0229	±0.0140
(*diff.*)	95% CI	0.0504	0.0920	0.0113	0.1183	0.0390	0.0613	0.0281	0.1449	0.0007	0.0451
	median	0.0640		0.0797		0.0432		0.0865		0.0211	
	min−max	0.0004	0.2350	0.0009	0.1210	0.0015	0.1514	0.0819	0.0911	0.0090	0.0406
Step length (m)	mean ± SD	0.5864	±0.0569 **	0.6599	±0.0618	0.6133	±0.0423	0.5877	±0.0332	0.6164	±0.0111
intact limb	95% CI	0.5665	0.6062	0.5950	0.7248	0.6012	0.6255	0.2898	0.8856	0.5988	0.6340
	median	0.5926		0.6542		0.6088		0.5877		0.6134	
	min−max	0.4563	0.6970	0.5897	0.7444	0.5231	0.7359	0.5642	0.6111	0.6071	0.6315
Step length (m)	mean ± SD	0.6121	±0.0502	0.6231	±0.0426	0.6123	±0.0554	0.6112	±0.0121	0.6172	±0.0353
amp. limb	95% CI	0.5946	0.6297	0.5784	0.6677	0.5964	0.6282	0.5023	0.7201	0.5611	0.6733
	median	0.6174		0.6435		0.6103		0.6112		0.6146	
	min−max	0.5176	0.7017	0.5437	0.6515	0.5001	0.7749	0.6027	0.6198	0.5868	0.6529
Step length (m)	mean ± SD	0.0627	±0.0533	0.0707	±0.0699	0.0373	±0.0328	0.0320	±0.0333	0.0221	±0.0019
*diff.*	95% CI	0.0440	0.0813	−0.0027	0.1440	0.0278	0.0467	−0.2675	0.3316	0.0191	0.0252
	median	0.0491		0.0488		0.0289		0.0320		0.0219	
	min−max	0.0016	0.2053	0.0039	0.1735	0.0007	0.1447	0.0084	0.0556	0.0201	0.0246

Abbreviations: TFA: transfemoral amputation, KDA: knee disarticulation, TTA: transtibial amputation, FA: foot amputation, NORM: normal gait (control group); *amp. limb*: amputated limb, *diff.*: difference between amputated and intact limb (correspondingly left/right limb in able-bodied); Note: *walking speed*: describes how fast a person moves in m/min; *cadence*: number of steps per minute; *step width*: distance between the heels of the two feet during double stance; *stance time*: time between the initial contact (IC) and the following toe-off (TO) of the same foot (time during which the foot has ground contact); *weight-acceptance peak* and *push-off peak*: the two characteristic peaks of the typically M-shaped vertical component of the GRF, the first taking place at the transition from loading response to mid stance, the second during terminal stance; *step length*: the spatial distance from the IC of one foot to the IC of the contralateral foot. Significant differences between younger (<60 years) and older (>60 years) patients are indicated by ** *p* < 0.001. Note that statistical analyses were only performed for the groups of TFA and TTA.

## Data Availability

All de-identified data will be shared on reasonable request immediately following publication. Researchers who provide a methodologically sound proposal may send their request to one of the corresponding authors.

## References

[1] Isakov E., Keren O., Benjuya N. (2000). Trans-tibial amputee gait: Time-distance parameters and EMG activity. Prosthet. Orthot. Int..

[2] Jaegers S.M., Arendzen J.H., de Jongh H.J. (1995). Prosthetic gait of unilateral transfemoral amputees: A kinematic study. Arch. Phys. Med. Rehabil..

[3] Bateni H., Olney S. (2002). Kinematic and Kinetic Variations of Below-Knee Amputee Gait. JPO J. Prosthet. Orthot..

[4] Nolan L., Wit A., Dudziñski K., Lees A., Lake M., Wychowañski M. (2003). Adjustments in gait symmetry with walking speed in trans-femoral and trans-tibial amputees. Gait Posture.

[5] Hof A.L., van Bockel R.M., Schoppen T., Postema K. (2007). Control of lateral balance in walking. Experimental findings in normal subjects and above-knee amputees. Gait Posture.

[6] Kovac I., Medved V., Ostojić L. (2010). Spatial, temporal and kinematic characteristics of traumatic transtibial amputees’ gait. Coll. Antropol..

[7] Highsmith M.J., Kahle J.T., Bongiorni D.R., Sutton B.S., Groer S., Kaufman K.R. (2010). Safety, energy efficiency, and cost efficacy of the C-Leg for transfemoral amputees: A review of the literature. Prosthet. Orthot. Int..

[8] Schaarschmidt M., Lipfert S.W., Meier-Gratz C., Scholle H.C., Seyfarth A. (2012). Functional gait asymmetry of unilateral transfemoral amputees. Hum. Mov. Sci..

[9] Roerdink M., Roeles S., van der Pas S.C., Bosboom O., Beek P.J. (2012). Evaluating asymmetry in prosthetic gait with step-length asymmetry alone is flawed. Gait Posture.

[10] Bell J.C., Wolf E.J., Schnall B.L., Tis J.E., Tis L.L., Potter B.K. (2013). Transfemoral amputations: The effect of residual limb length and orientation on gait analysis outcome measures. J. Bone Jt. Surg. Am. Vol..

[11] Castro M.P.D., Meucci M., Soares D.P., Fonseca P., Borgonovo-Santos M., Sousa F., Machado L., Vilas-Boas J.P. (2014). Accuracy and Repeatability of the Gait Analysis by the WalkinSense System. BioMed Res. Int..

[12] Uchytil J., Jandacka D., Zahradnik D., Farana R., Janura M. (2014). Temporal-spatial parameters of gait in transfemoral amputees: Comparison of bionic and mechanically passive knee joints. Prosthet. Orthot. Int..

[13] Wezenberg D., Cutti A.G., Bruno A., Houdijk H. (2014). Differentiation between solid-ankle cushioned heel and energy storage and return prosthetic foot based on step-to-step transition cost. J. Rehabil. Res. Dev..

[14] Adamczyk P.G., Kuo A.D. (2015). Mechanisms of Gait Asymmetry Due to Push-Off Deficiency in Unilateral Amputees. IEEE Trans. Neural Syst. Rehabil. Eng. Publ. IEEE Eng. Med. Biol. Soc..

[15] Cutti A.G., Verni G., Migliore G.L., Amoresano A., Raggi M. (2018). Reference values for gait temporal and loading symmetry of lower-limb amputees can help in refocusing rehabilitation targets. J. Neuroeng. Rehabil..

[16] Rutkowska-Kucharska A., Kowal M., Winiarski S. (2018). Relationship between Asymmetry of Gait and Muscle Torque in Patients after Unilateral Transfemoral Amputation. Appl. Bionics Biomech..

[17] Loiret I., Villa C., Dauriac B., Bonnet X., Martinet N., Paysant J., Pillet H. (2019). Are wearable insoles a validated tool for quantifying transfemoral amputee gait asymmetry?. Prosthet. Orthot. Int..

[18] Varrecchia T., Serrao M., Rinaldi M., Ranavolo A., Conforto S., De Marchis C., Simonetti A., Poni I., Castellano S., Silvetti A. (2019). Common and specific gait patterns in people with varying anatomical levels of lower limb amputation and different prosthetic components. Hum. Mov. Sci..

[19] Sanderson D.J., Martin P.E. (1997). Lower extremity kinematic and kinetic adaptations in unilateral below-knee amputees during walking. Gait Posture.

[20] Mattes S.J., Martin P.E., Royer T.D. (2000). Walking symmetry and energy cost in persons with unilateral transtibial amputations: Matching prosthetic and intact limb inertial properties. Arch. Phys. Med. Rehabil..

[21] Castro M.P., Soares D., Mendes E., Machado L. (2014). Plantar pressures and ground reaction forces during walking of individuals with unilateral transfemoral amputation. PM R J. Inj. Funct. Rehabil..

[22] Barr J.B., Wutzke C.J., Threlkeld A.J. (2012). Longitudinal gait analysis of a person with a transfemoral amputation using three different prosthetic knee/foot pairs. Physiother. Theory Pract..

[23] Hak L., van Dieën J.H., van der Wurff P., Houdijk H. (2014). Stepping asymmetry among individuals with unilateral transtibial limb loss might be functional in terms of gait stability. Phys. Ther..

[24] Jaegers S.M., Arendzen J.H., de Jongh H.J. (1996). An electromyographic study of the hip muscles of transfemoral amputees in walking. Clin. Orthop. Relat. Res..

[25] Hermodsson Y., Ekdahl C., Persson B.M., Roxendal G. (1994). Standing balance in trans-tibial amputees following vascular disease or trauma: A comparative study with healthy subjects. Prosthet. Orthot. Int..

[26] Goujon H., Bonnet X., Sautreuil P., Maurisset M., Darmon L., Fode P., Lavaste F. (2006). A functional evaluation of prosthetic foot kinematics during lower-limb amputee gait. Prosthet. Orthot. Int..

[27] Baum B.S., Schnall B.L., Tis J.E., Lipton J.S. (2008). Correlation of residual limb length and gait parameters in amputees. Injury.

[28] Chow D.H., Holmes A.D., Lee C.K., Sin S.W. (2006). The effect of prosthesis alignment on the symmetry of gait in subjects with unilateral transtibial amputation. Prosthet. Orthot. Int..

[29] Asano M., Rushton P., Miller W.C., Deathe B.A. (2008). Predictors of quality of life among individuals who have a lower limb amputation. Prosthet. Orthot. Int..

[30] Gailey R., Allen K., Castles J., Kucharik J., Roeder M. (2008). Review of secondary physical conditions associated with lower-limb amputation and long-term prosthesis use. J. Rehabil. Res. Dev..

[31] Morgenroth D.C., Gellhorn A.C., Suri P. (2012). Osteoarthritis in the Disabled Population: A Mechanical Perspective. PMR.

[32] Casey L.P., Nicholas F.T., Nora S. (2013). Patients receiving inpatient rehabilitation for lower limb orthopaedic conditions do much less physical activity than recommended in guidelines for healthy older adults: An observational study. J. Physiother..

[33] Struyf P.A., van Heugten C.M., Hitters M.W., Smeets R.J. (2009). The prevalence of osteoarthritis of the intact hip and knee among traumatic leg amputees. Arch. Phys. Med. Rehabil..

[34] Winter D.A., Sienko S.E. (1988). Biomechanics of below-knee amputee gait. J. Biomech..

[35] Engsberg J.R., Lee A.G., Tedford K.G., Harder J.A. (1993). Normative ground reaction force data for able-bodied and below-knee-amputee children during walking. J. Pediatr. Orthop..

[36] Sagawa Y., Turcot K., Armand S., Thevenon A., Vuillerme N., Watelain E. (2011). Biomechanics and physiological parameters during gait in lower-limb amputees: A systematic review. Gait Posture.

[37] Kastner J.W.P. (1997). Angewandte Ganganalyse in der Rehabilitation. Med. Orth. Tech. Gentner Verl. Stuttg..

[38] Perry J., Bowker J., Surgeons A. (2002). Normal Gait. Atlas of Limb Prosthetics: Surgical, Prosthetic, and Rehabilitation Principles.

[39] Beck R.J., Andriacchi T.P., Kuo K.N., Fermier R.W., Galante J.O. (1981). Changes in the gait patterns of growing children. J. Bone Jt. Surg. Am. Vol..

[40] Aloba A., Luc A., Woodward J., Dong Y., Zhang R., Jain E., Anthony L. Quantifying Differences Between Child and Adult Motion Based on Gait Features. Proceedings of the Universal Access in Human-Computer Interaction. Multimodality and Assistive Environments.

[41] Bieringer S., Sibbel B., Kokegei D. (2007). Exoskelettale Prothesen der unteren Extremität. Orthopädie Und Unf. Up2date.

[42] Greitemann B. (2016). Amputationen am Fuß. Orthopädie Und Unf. Up2date.

[43] Greitemann B., Brückner L., Schäfer M., Baumgartner R. (2016). Amputation und Prothesenversorgung.

[44] Behr J., Friedly J., Molton I., Morgenroth D., Jensen M.P., Smith D.G. (2009). Pain and pain-related interference in adults with lower-limb amputation: Comparison of knee-disarticulation, transtibial, and transfemoral surgical sites. J. Rehabil. Res. Dev..

[45] Caroline E.R., John B., Garry T.A. (2014). Predictors of non-use of prostheses by people with lower limb amputation after discharge from rehabilitation: Development and validation of clinical prediction rules. J. Physiother..

